# Case report: Surgical strategies of a giant thrombus from the ascending aorta to the arch

**DOI:** 10.3389/fcvm.2023.1091303

**Published:** 2023-02-24

**Authors:** Guanhua Li, Yingzhen Chen, Haikuo Wang, Yanping Liu, Hangyu Liu, He Sun, Zhiping Wang

**Affiliations:** ^1^Department of Cardiovascular Surgery, Sun Yat-sen Memorial Hospital, Sun Yat-sen University, Guangzhou, Guangdong, China; ^2^Department of Anesthesiology, Sun Yat-sen Memorial Hospital, Sun Yat-sen University, Guangzhou, Guangdong, China; ^3^Department of Hepatobiliary Surgery, Sun Yat-sen Memorial Hospital, Sun Yat-sen University, Guangzhou, Guangdong, China; ^4^Department of Pathology, Sun Yat-sen Memorial Hospital, Sun Yat-sen University, Guangzhou, Guangdong, China

**Keywords:** thrombus, ascending aorta, aortic arch, surgical strategies, deep hypothermic circulatory arrest

## Abstract

Large mural thrombi in the relatively normal ascending aorta are extremely uncommon conditions that may lead to major adverse cardiovascular events due to new embolism. Because of their changeable variations, the management of these unstable thrombi is challenging and controversial. The size, morphology, location, embolic involvement, and patients’ conditions are all crucial for therapeutic decision-making. Treatment options include anticoagulation, thrombolysis, surgical thrombectomy, and endovascular stenting. Therefore, surgical strategies should be highly individualized. Herein, we present a rare case of a huge thrombus from the ascending aorta to the arch in a 43-year-old man. Considering the high risks of catastrophic embolic events, surgical removal of the aortic mass, thromboendarterectomy, and reconstruction of the arterial wall were performed with a satisfactory outcome. This report illustrates our experience of surgical strategies and perioperative treatments for this challenging case, and contemporary surgical management for mural thrombi in the ascending aorta was also thoroughly discussed.

## Introduction

Non-atherosclerotic and non-aneurysmal thrombi located in the ascending aorta are scarce since the high-speed flow environment would have suppressed thrombotic formation (1). It has the possibility of catastrophic complications due to secondary embolism, including stroke, myocardial infarction (2), and peripheral embolism (3, 4). The treatment for thrombi in the normal ascending aorta is challenging and should be in an individualized manner (5). Herein, we present a rare case of a huge thrombus from the ascending aorta to the arch, which was surgically removed with a satisfactory outcome.

## Case presentation

**Figure 1:**
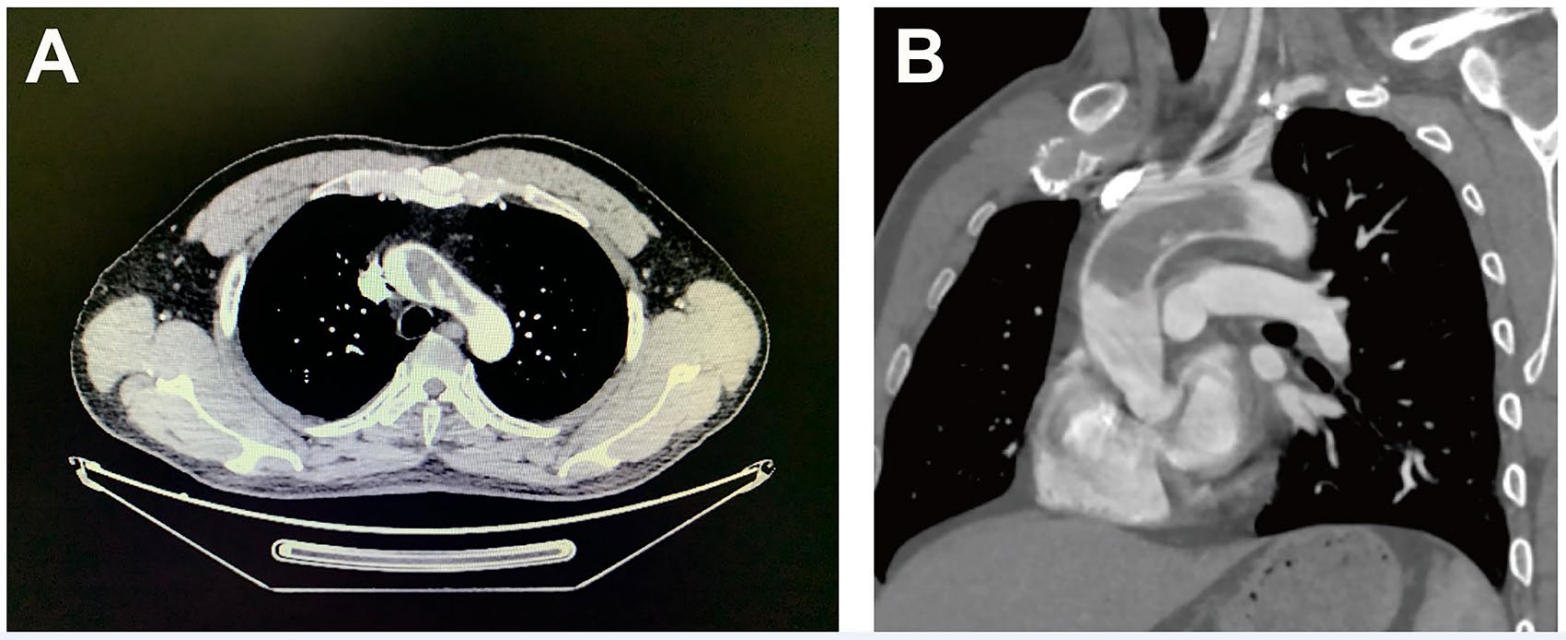
Computer tomography images (cross-section for Panel **A** and longitudinal-section for Panel **B**) of a giant floating thrombus from the ascending aorta to the arch.

A 43-year-old man with a history of stroke was referred to our department due to a giant mass located in the aorta found incidentally in a local hospital. The patient presented with slurred speech and paralysis of the right limb. No histories of atherosclerosis, coronary artery diseases, hypertension, and trauma were noted. No symptoms of coronary ischemia or thrombosis of the extremities were found. The patient was a non-smoker and was healthy previously with no medications. The contrast-enhanced computer tomography of the whole aorta revealed a low-density lesion without enhancement from the ascending aorta to the arch and ruled out the possibility of malignancy (Figures 1A,B). The results of positron-emission tomography, tumor biomarkers, inflammatory biomarkers, and clotting tests were also normal (Table 1). The biological hemostasis tests, including mutation detection for factor II and factor V, levels of antithrombin III, protein C, protein S, and anti-phospholipid antibodies were within normal ranges. The coronavirus disease 2019 (COVID-19) test was negative. Atrial fibrillation was not detected in the electrocardiogram, and normal cardiac function was found in ultrasonography, without the presence of an intraventricular thrombus, cardiac tumor, patent foramen ovale, and concomitant deep vein thrombosis. Considering the risks for secondary embolic events and myocardial infarction of the floating mass, surgery involving the removal of the aortic mass and thromboendarterectomy was indicated. However, a large infarct area in the left cerebral hemisphere with scattered hemorrhagic lesions was observed on admission by the cerebral computed tomography, and surgery was therefore postponed for 3 weeks (Supplementary Figure S1).

**Table 1:** Clinical characteristics of the patient on admission.

Clinical characteristics	
Age	43
Gender	Male
BMI	25.1 kg/m^2^
Red blood cell	4.56*10^12/L
Hemoglobin	146 g/L
White blood cell	7.38*10^9/L
Proportion of neutrophils	54.5%
Platelet	157*10^9/L
PTINT	0.96
PT	11.4S
D-dimer	0.98 mg/l FEU↑
TNF	0.50 pg./mL
Interferon	0.43 pg./mL
Interleukin 6	0.78 pg./mL
cTnT	6.1 pg./mL
NT-proBNP	18.3 pg./mL
ESR	7.0 mm/h
rheumatoid factors	<11.3
Antistreptolysin O	68.5 IU/mL
Triglycerides	1.70 mmol/L
Albumin	41.9 g/L
Total cholesterol	4.19 mmol/L
HDL cholesterol	0.68 mmol/L
LDL cholesterol	2.62 mmol/L

PTINT, Prothrombin international ratio; PT, Prothrombin time; TNF, tumor necrosis factor; ESR, Erythrocyte sedimentation rate.

**Figure 2:**
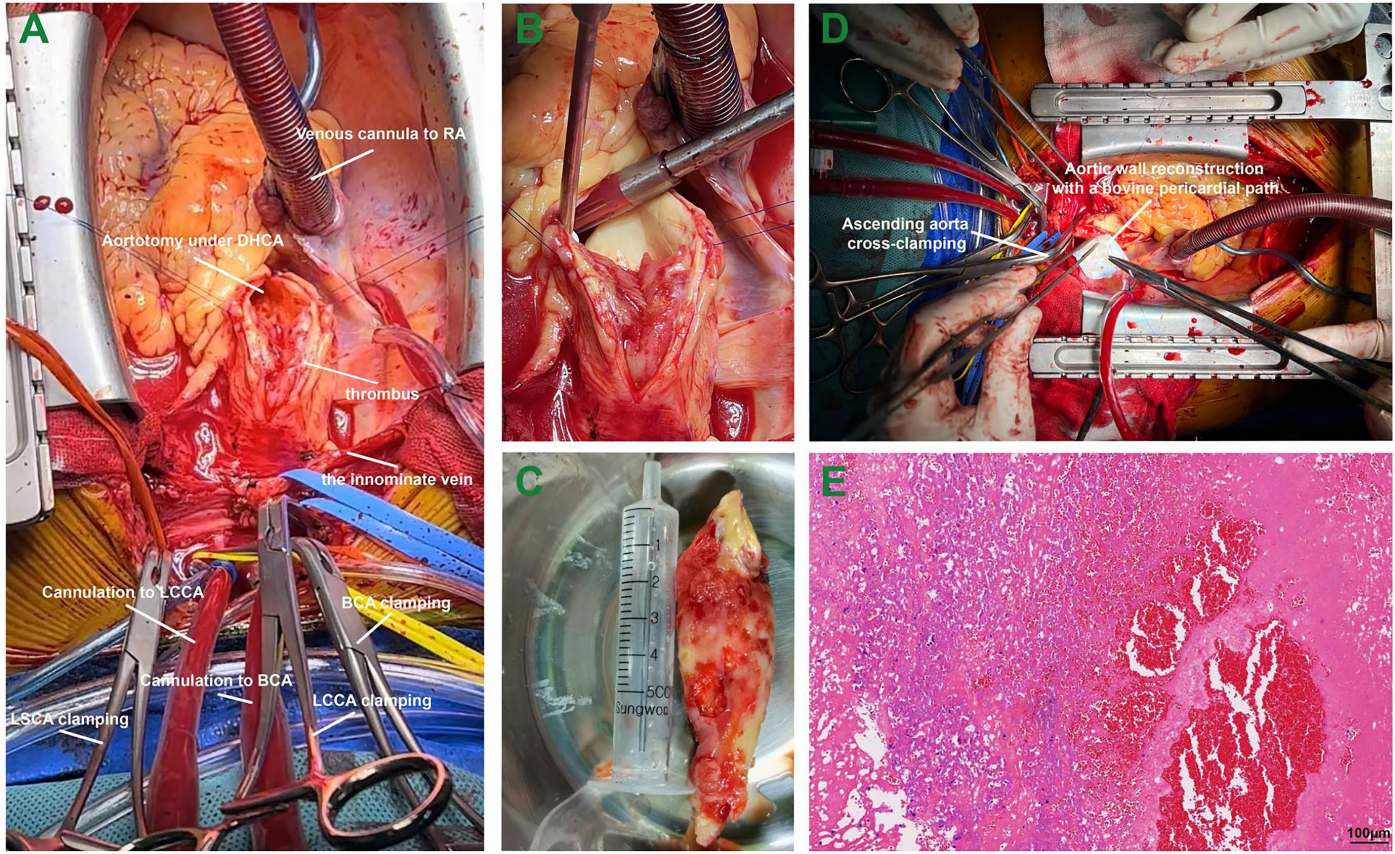
The surgical procedure in a patient with a giant thrombus from the ascending aorta to the arch. Panel **A**, the cannulation strategies for bilateral cerebral perfusion; Panel **B**, the giant floating thrombus attached to the aortic wall of the ascending aorta; Panel **C**, the giant thrombus measuring 9*4 cm in size; Panel **D**, the aortic wall reconstruction using a bovine pericardial patch; and Panel **E**, pathological evaluation of the thrombus in hematoxylin and eosin staining. Abbreviations: RA, right atrium; DHCA, deep hypothermic circulatory arrest; BCA, brachiocephalic artery; LCCA, left common carotid artery; LSCA, left subclavian artery.

At 3 weeks after admission, surgery was performed *via* median sternotomy, with all supra-aortic branches mobilized. The femoral artery was used to perfuse the body, while the brachiocephalic artery and the left common carotid artery were cannulated for bilateral cerebral perfusion (Figure 2A). With a venous cannula placed in the right atrium, the cardiopulmonary bypass was established in a pulsatile fashion (6). Cooling to 26°C, the cardiac rhythm turned to ventricular fibrillation. To avoid secondary embolism, all supra-aortic branches were clamped with the circuit flow decreased to 10%. We removed the floating mass *via* aortotomy without aortic cross-clamping, which was 9*4 cm in size and was attached to the wall of the ascending aorta (Figures 2B,C). The cardioplegic solution was delivered through the coronary ostia, and the heart was arrested successfully. The ascending aorta was clamped subsequently, and the rewarming process was started. The site of attachment was further excised, and the wall of the ascending aorta was reconstructed using a bovine pericardial patch measuring 5*5 cm in size (Figure 2D). Pathological examination confirmed that the red-yellow mass was a thrombus (Figure 2E). The presence of fibrosis and infiltration of neutrophils suggested that the thrombus was formed several months ago (7). Neither atherosclerotic plaque nor endothelial injury was seen in the thrombus-aortic wall attachment zone (Supplementary Figure S2). Postoperative hemodynamics went smoothly, without the usage of inotropes. Glucocorticoids and antibiotics were prophylactically administered for 5 days postoperatively. With long-term anticoagulants administered, the patient was discharged 12 days after surgery. The patient returned to a local rehabilitation hospital for stroke recovery. The movement and mobility of the body are now greatly improved, with normal flexibility for speech after 6 months of follow-up.

## Discussion and conclusion

Non-atherosclerotic and non-aneurysmal thrombi in the ascending aorta are scarce due to the high-speed flow environment (1). In the present case, the etiology of this giant thrombus raises our great concerns. The arterial wall implant site and the peduncle of the thrombus were carefully examined, while histopathology revealed no evidence of atherosclerosis and other lesions. We also observed sporadic lymphocytic infiltration and fibrotic transformation in the mass, suggesting that the thrombus was formed for months. Amid the COVID-19 pandemic, the cytokine storm and hypoxemia caused by COVID-19 may increase the risk for arterial thrombosis (8), and a recent study also revealed that COVID-19 might attack the vascular endothelium, resulting in endothelial injury (9). However, the test for COVID-19 was negative in this patient. The mechanism of thrombosis, in this case, is still unclear; however, regional weakness and inflammation of the endothelium may contribute to thrombosis.

Before considering surgical interventions, it is crucial to exclude some conditions such as malignancy, vasculitis, and hypercoagulation (10); hence, results of positron-emission tomography, tumor biomarkers, inflammatory biomarkers, and clotting tests must be obtained before decision-making. Verma et al. (5) classified mural thrombi of the aorta into four types (type I: the ascending aorta and the arch until the left subclavian artery origin; type II: the descending thoracic aorta up to the celiac artery; type III: between the celiac artery and the most distal renal artery; and type IV: between the lowest renal artery and the aortic bifurcation) based on the location. Type I thrombi, as this case presented, are the least common. According to morphology, aortic thrombi could be sessile, pedunculated, or occlusive. Karalis et al. (11) reported that much more embolic events occur in the pedunculated thrombi as compared to other thrombi with non-floating features. A systematic review comparing anticoagulation only and surgical treatment for aortic mural thrombus reported that the recurrence rate and complication rate are significantly lower in patients who were surgically managed (12).

Currently, there are no guidelines or therapeutic consensus on the management of aortic thrombi (13). Therapeutic options include anticoagulant medications, thrombolysis, surgical thrombectomy, endovascular stenting, and balloon embolectomy, which should be used in an individualized manner based on size, location, morphology, and involvement (5). For typically type I thrombi, especially those with pedunculated or floating features, which have higher risks for embolic events, surgical thrombectomy on cardiopulmonary bypass should be indicated. If supra-aortic branches were involved, embolectomy or supra-aortic debranching procedure with stent implantation should be considered (5). Arterial cannulation strategies for cardiopulmonary bypass in type I thrombi should also be individualized to ensure the best visceral or cerebral perfusion and the lowest risks for exfoliation of the thrombus. Sites for cannulation include the femoral artery, axillary artery, and other supra-aortic branches.

Considering the relatively large size, floating characteristics, and the previous history of embolic events in this case, surgical removal of the thrombus and aortic wall reconstruction were performed under cardiopulmonary bypass with deep hypothermic circulatory arrest. It is noteworthy that circulatory arrested should be used if possible clamping sites were unavailable because of thrombotic involvement. Bilateral cerebral perfusion was achieved by dual cannulation of supra-aortic branches, while the retrograde flow of femoral cannulation not only perfused the body but also prevented secondary distal embolism caused by small thrombotic fragments.

It is recommended that the site of thrombus implantation should be surgically excised or replaced to prevent occurrence (7). We used a bovine pericardial patch to reconstruct the arterial wall in this case; however, segmental aortic replacement should be indicated in those sessile thrombi with larger attachment sites. Long-term aspirin was administered postoperatively to avoid recurrence owing to the possible residual thrombus (14). In brief, although guidelines or consensus on the treatments for thrombi located in the ascending aorta and the arch, surgical removal is strongly recommended in those pedunculated or floating thrombi that carry higher risks for secondary embolic events.

## Data availability statement

The raw data supporting the conclusions of this article will be made available by the authors, without undue reservation.

## Ethics statement

The studies involving human participants were reviewed and approved by the Ethics Committee of Sun Yat-sen Memorial Hospital. The patients/participants provided their written informed consent to participate in this study. Written informed consent was obtained for the publication of this case report.

## Author contributions

GL and HW prepared and wrote the manuscript. YC and YL prepared surgical and pathological figures. HL and HS collected and analyzed the patient’s data. ZW reviewed and revised the manuscript. All authors have read and approved the manuscript and agreed to be accountable for the content of the manuscript.

## Funding

This study was supported by grants from the Medical Scientific Research Foundation of Guangdong Province of China (grant no. A2021433) and Guangdong Basic and Applied Basic Research Foundation (grant no. 2021A1515010280 and 2022A1515012170).

## Conflict of interest

The authors declare that the research was conducted in the absence of any commercial or financial relationships that could be construed as a potential conflict of interest.

## Publisher’s note

All claims expressed in this article are solely those of the authors and do not necessarily represent those of their affiliated organizations, or those of the publisher, the editors and the reviewers. Any product that may be evaluated in this article, or claim that may be made by its manufacturer, is not guaranteed or endorsed by the publisher.

## Supplementary material

The Supplementary material for this article can be found online at: https://www.frontiersin.org/articles/10.3389/fcvm.2023.1091303/full#supplementary-material
