# Supplementary figures and images for: Case report: Surgical strategies of a giant thrombus from the ascending aorta to the arch
Source: Front Cardiovasc Med. 2023 Feb 24;10:1091303. doi: 10.3389/fcvm.2023.1091303 (PMC9996118; doi:10.3389/fcvm.2023.1091303)

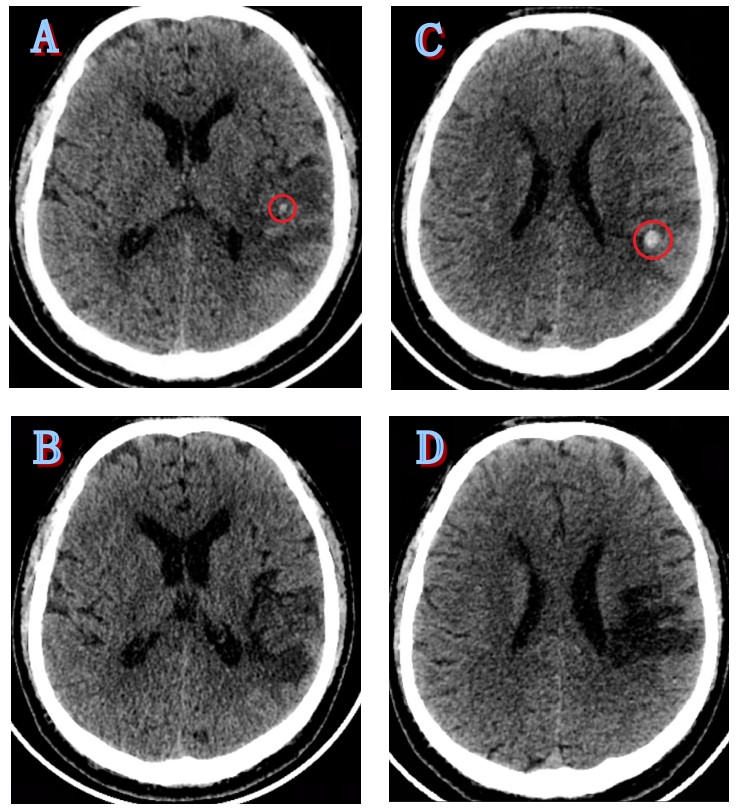

Supplement: Supplementary file 1 [file Image_1.JPEG]

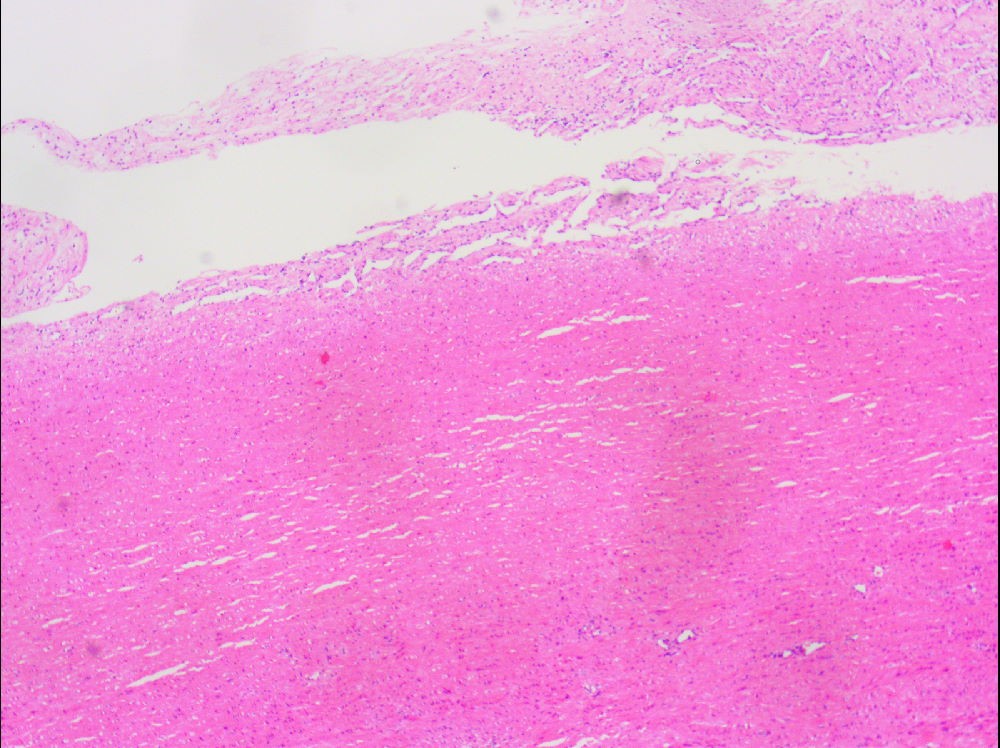

Supplement: Supplementary file 2 [file Image_2.JPEG]
